# Evaluation of prevalence's of *pfdhfr *and *pfdhps *mutations in Angola

**DOI:** 10.1186/1475-2875-10-22

**Published:** 2011-02-02

**Authors:** Filomeno Fortes, Rafael Dimbu, Paula Figueiredo, Zoraima Neto, Virgílio E do Rosário, Dinora Lopes

**Affiliations:** 1 Programa Nacional de Controlo da Malária/Ministério da Saúde de Angola, Luanda, Angola; 2 UEI Malária/Centro de Malária e Doenças Tropicais - LA/IHMT/Universidade Nova de Lisboa, Rua da Junqueira, 100, 1349-008, Lisboa, Portugal; 3 Health Progress and Investigation Network of Portuguese-Speaking Countries Community (RIDES/CPLP), Centro de Malária e Doenças Tropicais - LA, Instituto de Higiene e Medicina Tropical, Universidade Nova de Lisboa, Lisboa, Portugal

## Abstract

**Background:**

Malaria is the major cause of morbidity and mortality in Angola. The most vulnerable groups to *Plasmodium falciparum *infection are pregnant women and children under five years of age. The use of an intermittent preventive treatment (IPT) with sulphadoxine/pyrimethamine (SP) in pregnant women was introduced in Angola in 2006 by the National Malaria Control Programme, and currently this strategy has been considered to be used for children malaria control. Considering the previous wide use of SP combination in Angola, together to the reported cases of SP treatment failure it is crucial the evaluation of the prevalence of five mutations in *pfdhfr *and *pfdhps *genes associated to *P*. *falciparum *resistance to SP before the introduction of S/P IPT in children.

**Methods:**

The study was conducted in five provinces, with different transmission intensities: Huambo, Cabinda, Uíge, Kwanza Norte, and Malanje. The detection of the mutations in *pfdhfr *and *pfdhps *genes was carried out in 452 *P*. *falciparum *blood samples by PCR RFLP.

**Results:**

For *pfdhfr *gene, 90,3% of the samples carried the mutation 51**I**, with 7.5% of mixed infections; 51% carried wild type allele 59**C**, with 29.2% mixed infections and; 99.1% of isolates harboured the mutant allele 108**N**. Concerning, *pfdhps *gene, 83,1% were mutant type 437**G **with 11% mixed infections , while 87% of the studied isolates were wild type for codon 540.

**Discussion:**

This is the first representative epidemiological study of the whole Angola country on the prevalence of the genotypes associated with SP chemoresistance. A high frequency of individual mutations in both genes (51**I **and 108**N **in *pfdhfr*, and 437**G **in *pfdhps*) was found, besides a low prevalence of the quintuple mutation.

**Conclusion:**

The data showed that the implementation IPT using SP in children needs to be reviewed.

## Background

According to the Angolan National Malaria Control Programme (NMCP), malaria is the major cause of morbidity and mortality in Angola, with four million clinical cases and 20 thousand deaths reported in 2005, accounting for 35% of the overall mortality in children under five years old and 25% of the maternal deaths [1-3]. Malaria is endemic throughout the Angolan territory, *Plasmodium falciparum *being the predominant species [4]. Due to the high prevalence of *P*. *falciparum *strains resistant to chloroquine [5-8], therapeutic regimens for treatment of uncomplicated *P. falciparum *infection were changed in 2006 [9] and, currently, the first-line treatment for uncomplicated malaria is Coartem^® ^(artemether-lumefantrine) followed by the amodiaquine-artesunate alternative therapy.

Additionally, in all Angolan endemic areas the strategy to protect mothers during their pregnancy includes the use of an intermittent preventive treatment (IPT) [10,11]. This intervention has been introduced in Angola since 2006, using sulphadoxine/pyrimethamine (SP) at the second trimester of pregnancy.

In other African countries, SP - ITP has been also introduced in children as a control measure to reduce malaria morbidity and mortality in this most vulnerable population [12] and has been evaluated in a number of clinical trials in these countries, with success [12-17]. Thus, Angolan NMCP intends in the near future to introduce this control measure in Angola. However, due to the wide use of SP combination in this country together to reported cases of SP treatment failure, it was decided to obtain further information about SP resistance in Angola, during a surveillance study carried out in 2007, before the introduction of such a control measure.

It is well known that mutations at the dihydropteroate synthase (*pfdhps*) and dihydrofolate reductase (*pfdhfr*) genes are associated with resistance of *P. falciparum *to SP, respectively [18-21]. In *pfdhfr*, point mutations at positions 51, 59, 108, and 164 are associated with pyrimethamine resistance [22,23]. Similarly, mutations in codons 437 (437G) and 540 (540E) of *pfdhps *are associated with resistance to sulphadoxine [24-29].

Thus, to determine the polymorphism of *pfdhps *and *pfdhfr *genes, infected blood samples were collected in different representative endemic regions of the whole country (Uíge, Kwanza Norte, Malanje, Cabinda and Huambo) and the prevalence of five mutations of the *pfdhfr *(N51I, C59R and S108N) and *pfdhps *(A437G and K540E) genes was investigated.

## Methods

### Sample collection and DNA extraction

**Figure 1:**
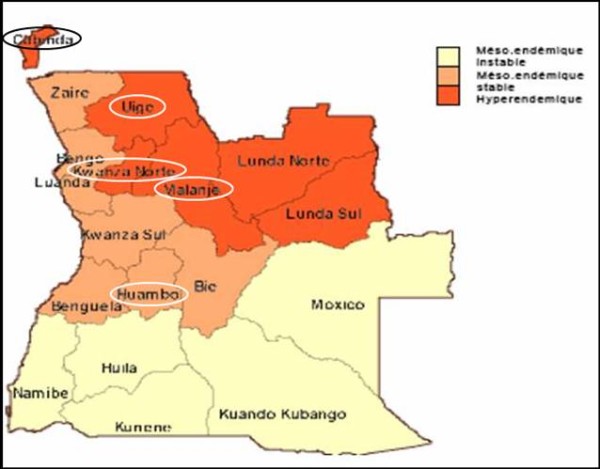
**Angola Map (adapted from http://www.rollbackmalaria.org/countryaction/angola_mis.html); the circles indicate the provinces of study**.

The blood samples used for this study were collected originally as part of malaria surveillance activities of NMCP. Community-based surveys were conducted in five areas with different transmission intensity: Huambo, Cabinda, Uíge, Kwanza Norte, and Malanje (Figure 1). These samples were collected from asymptomatic children under five years of age, at time of blood collection. Each sample consisted of 200 μl of finger-prick blood spotted on filter papers, dried and stored at room temperature until parasite DNA extraction.

Parent's informed consent was obtained before inclusion in the study, which was reviewed and approved by the Ethical Committee from the Ministry of Health of Angola. DNA was extracted from blood spotted on filter paper, using phenol-chloroform method as described elsewhere [30].

### Analysis of mutations

Polymorphisms on the codons 51, 59, 108 in *pfdhfr *and 437, 540 in *pfdhps *were assessed by PCR RFLP after amplification of DNA fragments by nested-PCR. The PCR and enzymes restrictions reactions were carried out under the conditions already described [31,32]. PCR amplicons and digested fragments were separated on 2% or 3% agarose gels stained with ethidium bromide and visualized under UV.

## Results

From one thousand and twelve samples collected in five provinces from children that did not exhibit malaria symptoms at the time of blood collection, 452 *P. falciparum *PCR positive samples were analysed in this study: 208 (46%) were collected in Malanje, 96 (21%) in Kwanza Norte, 71 (16%) in Cabinda, 54 (12%) in Uíge and 23 (5%) in Huambo provinces.

For *pfdhfr *gene, all 452 samples were successfully characterized by PCR-RFLP for 51, 423 and 59 codons and 430 samples for codon 108. Regarding *pfdhps *gene, 438 samples were characterized for codon 437 and 448 samples for codon 540. The analysis of *pfdhfr *showed that 90,3% (408 out 452) of the isolates carried the mutant allele 51**I**, while 7,5% (34 out 452) carried a mixed infection (**N **and **I**); for 59 codon 51% (213 out 423) were wild type (C59), 29,2% (122 out 423) were mixed infections (**C **and **R**) and 19,9% (83 out 423) carried the mutant allele 59**R**. Concerning the *pfdhfr *gene codon 108, 99,1% (426 out 430) of isolates harbored the mutant allele (**N**). For *pfdhps *83,1% (364 out 438) were mutant type (437**G**), 11% (48 out 438) were mixed populations and 87% (390 out 448) of studied isolates were wild type for codon 540 (**K**) (Table 1).

**Table 1:** Prevalence of mutations conferring resistance to SP in *P. falciparum *isolates from Angola.

Prevalence of mutations in *pfdhfr *and *pfdhps *genes (%)						
		***pfdhfr***			***pfdhps***	
			
**Province**		**51**	**59**	**108**	**437**	**540**
	**Alleles**	**n (%)**	**n (%)**	**n (%)**	**n (%)**	**n (%)**

	**wild type**	0 (0)	16 (27,6)	0 (0)	8 (12,1)	55 (83,3)
**Cabinda**	**mutant**	70 (98,6)	28 (48,3)	64 (100)	57 (86,4)	3 (4,5)
	**mix infection**	1 (1,4)	14 (24,1)	0 (0)	1 (1,5)	8 (12,1)
	**wild type**	1(1,85)	19 (35,8)	0(0)	0 (0)	53 (98,1)
**Uige**	**mutant**	52 (96,3)	15 (28,3)	50 (100)	52 (96,3)	1 (1,9)
	**mix infection**	1 (1,85)	19 (35,8)	0 (0)	2 (3,7)	0 (0)
	**wild type**	2 (2,1)	46 (49,5)	0 (0)	0 (0)	92 (95,8)
**Kwanza Norte**	**mutant**	91 (94,8)	25 (26,9)	92 (100)	93 (96,9)	2 (2,1)
	**mix infection**	3 (3,1)	22 (23,7)	0 (0)	3 (3,1)	2 (2,1)
	**wild type**	7 (3,4)	127 (62,6)	3 (1,5)	16 (7,7)	189 (90,9)
**Malanje**	**mutant**	173 (83,2)	12 (5,9)	199 (98)	156 (75)	5 (2,4)
	**mix infection**	28 (13,5)	64 (31,5)	1 (0,5)	34 (16,3)	13 (6,3)
	**wild type**	0 (0)	5 (45,5)	0 (0)	2 (12,5)	11 (61,1)
**Huambo**	**mutant**	22 (95,7)	3 (27,3)	21 (100)	6 (37,5)	1 (5,6)
	**mix infection**	1 (4,4)	3 (27,3)	0 (0)	8 (50)	6 (33,3)
	**wild type**	10 (2,2)	213 (51,0)	3 (0,7)	26 (5,9)	390 (87,1)
**Total**	**mutant**	408 (90,3)	83 (19,9)	426 (99,1)	364 (83,1)	29 (6,5)
	**mix infection**	34 (7,5)	122 (29,2)	1 (0,2)	48 (11,0)	29 (6,5)

A mixture of infections with wild-type and mutant alleles was also observed. These mixed infections were seen for *pfdhfr *gene in positions 51 (34/452), 59 (122/416) and 108 (1/430), and in *pfdhps *gene in positions 437 (45/438) and 540 (29/441). All mixed infections were excluded from subsequent analysis. Therefore, successful characterization of all five molecular markers was obtained only in 241 samples out of a total of 452.

Only 25% (72) of the 400 isolates which were successfully characterized for the studied *pfdhfr *codons harboured the triple *pfdhfr *51-59-108 mutations and one isolate carried the combination of three wild type codons. The more frequent *pfdhfr *genotype was **ICN**, which was found in 200 isolates (Table 2). Regarding the *pfdhps *genotype **GK**, double mutation (437 and 540 codons) showed the highest frequency (91,1%). 225 isolates harboured mutations on position 108 of *pfdhfr *gene and 437 of *pfdhps *gene that were reported as the initial mutations for pyrimethamine and sulphadoxine resistance, respectively [23-36].

**Table 2:** Prevalence of haplotypes in *P. falciparum *isolates from Angola.

	Genotypes	Cabinda		Uige		Kwanza Norte		Malanje		Huambo		Total	
		n	(%)	n	**(%)**	n	(%)	n	**(%**)	n	**(%**)	n	**(%**)
	51 59 108												
	IRN	28	63,6	13	40,6	22	32,4	5	3,9	1	16,7	**69**	24,8
	ICN	16	36,4	18	56,3	44	64,7	117	91,4	5	83,3	**200**	71,9
***pfdhfr***	ICS		0,0		0,0		0,0	1	0,8		0,0	**1**	0,4
	NCN		0,0		0,0		0,0	2	1,6		0,0	**2**	0,7
	NRN		0,0	1	3,1	2	2,9	3	2,3		0,0	**6**	2,2
	**n**	44		32		68		128		6		278	
	437 540												
	GK	50	87,7	51	98,1	89	94,7	144	89,4	5	62,5	**339**	91,1
***pfdhps***	GE	3	5,3	1	1,9	2	2,1	5	3,1	1	12,5	**12**	3,2
	AK	4	7,0	0	0,0	3	3,2	12	7,5	2	25,0	**21**	5,6
	**n**	57		52		94		161		8		372	
	51 59 108/437 540												
	ICN/GK	10	27,8	17	54,8	41	60,3	83	81,4	1	25,0	**152**	63,1
	IRN/GK	22	61,1	14	45,2	20	29,4	4	3,9	1	25,0	**61**	25,3
	IRN/AK	2	5,6		0,0	2	2,9		0,0		0,0	**4**	1,7
	ICN/AK		0,0		0,0	1	1,5	6	5,9	2	50,0	**9**	3,7
	NRN/GK		0,0		0,0	2	2,9	1	1,0		0,0	**3**	1,2
***pfdhfr/pfdhps***	ICN/GE	1	2,8		0,0	2	2,9		0,0		0,0	**3**	1,2
	ICS/GK		0,0		0,0		0,0	1	1,0		0,0	**1**	0,4
	ICN/GE		0,0		0,0		0,0	4	3,9		0,0	**4**	1,7
	NCN/GK		0,0		0,0		0,0	1	1,0		0,0	**1**	0,4
	NRN/AK		0,0		0,0		0,0	1	1,0		0,0	**1**	0,4
	NCN/AK		0,0		0,0		0,0	1	1,0		0,0	**1**	0,4
	IRN/GE	1	2,8		0,0		0,0		0,0		0,0	**1**	0,4
	**n**	**36**		**31**		**68**		**102**		**4**		**241**	

In *pfdhfr *gene, five mutant genotypes, **NCN**, **NRN**, **ICN**, **IRN **and **NCS **(amino acids at positions 51, 59 and 108) were found confirming the major diversity of this gene (Table 2). Among the studied isolates, 74% were double mutants (**ICN **or **NRN**), most of them being type **ICN**, and the triple mutant **IRN **was detected in 25% of the samples. Only one isolate was a single mutant (**ICS**). In *pfdhps*, three allele combinations **GK**, **GE **and **AK **(amino acids at positions 437 and 540) were detected nearly 3% being the double mutant **GE **and 91% of the isolates were **GK **and 6% were wild-type (**AK**). Considering the two studied genes, 12 different genotype combinations were found: **NRN GK**, **ICN GK**, **IRN GK**, **IRN GE**, **ICN AK**, **IRN AK**, **ICN GE**, **NCN AK**, **NRN AK**, **ICS GK**, **NCN GK **and **NCS GK **(51, 59 and 108 for *pfdhfr *gene and 437 and 540 for *pfdhps *gene). From a total of 241 isolates, 63% were **ICN GK**, 25% **IRN GK**, 3,7% were **ICN AK**, **ICN GE**. **NRN GK **and **IRN AK **were detected with same frequency of the 1,7%, 1,2% were **NRN GK **and **ICN GE**, all other combinations were found with a very low frequency (Table 2).

In a comparison evaluation between provinces, the same pattern was found except for Cabinda, where the most frequent genotype was **IRN **(Tables 1 and 2).This province is geographically separated from the rest of the country.

## Discussion

The monitoring of SP resistance is relevant in order to guide national malaria treatment policies before introduction of SP as IPT in children at Angola. In this light, this study was designed to assess the *pfdhfr *and *pfdhps *mutations associated with SP chemoresistance. For this purpose, the mutations at *pfdhfr *(**N**51**I**, **C**59**R **and **S**108**N**) and *pfdhps *(**A**437**G **and **K**540**E**) genes, considered predictive of SP treatment failure [25-27], were assessed in five provinces of Angola. Four of them - Uíge, Kwanza Norte, Malanje and Cabinda - are hyperendemic areas, whereas Huambo, is a mesoendemic stable. It this way, it was observed the presence of the mutations 51**I **and 59**R **jointly with 108**N**, which enhances the level of resistance to pyrimethamine when compared with single mutation in codon 108 [35-37]. Similarly, mutations in 437 (437**G**) and 540 (540**E**) *pfdhps *codons are associated with resistance to sulphadoxine [24-29]. In *pfdhfr *gene, the mutation at position 108 (S108**N**), which is believed to be the initial mutation causing pyrimethamine resistance, was observed in almost all isolates successfully characterized for this codon (426/430) (Table 1). In *pfdhps *gene, among the 438 characterized isolates, 364 presented the mutant allele at position 437, which has been reported as the initial mutation for sulphadoxine resistance in many endemic regions.

From 430 *P*. *falciparum *isolates characterized in this study, 99,1% carried the *pfdhfr *108**N **mutation. The results also showed that 27% of *P*. *falciparum *isolates presented double mutations at codons 59 and 108, indicating the development of resistance against antifolates in Angola. Another double mutant (51**I **108**N**) was observed in 96,7% of Angolan *P*. *falciparum *isolates; these results were consistent with the results obtained in a similar study carried out at Uíge province in 2009 [38].

A 25% of *pfdhfr *triple mutant prevalence (51**I**/59**R**/108**N**) was also noticed. The prevalence of these mutations in association with high prevalence of mutation at position 437**G **in *pfdhps *(83,1%) may indicate that these *P*. *falciparum *parasite populations have the potential to evolve into *pfdhfr*/*pfdhps *quintuple mutant in the near future, a mutant that is considered a molecular marker of SP treatment failure [25-27].

In addition, the simultaneously presence of 59**R ***pfdhfr *and 540**E ***pfdhps *variants is considered predictive of the presence of quintuple mutant (*pfdhfr *51**I**, 59**R**, 108**N**, *pfdhps *437**G**, 540**E**) [25-40]. In fact, despite high frequencies of mutations in this report, only one isolate was found harbouring the quintuple mutant associated with high level of resistance to SP. This finding is in accordance with the results obtained in other similar studies carried out in Angola, Republic of Congo and Gabon [31,28,29].

The predominant *pfdhfr *haplotype in the present work was 51**I**59**C**108**N **(71,9%), corroborating the results reported with Brazilian samples by Gama and collaborators [41]. The triple mutant 51**I**59**R**108**N **(24,8%) was of low prevalence in the examined isolates, similarly to previous data reported in Sri Lanka [42] and Papua New Guinea [43], but different from the isolates from Malaysia [44], Brazil [45] and India [46] where this triple mutant was the predominant haplotype. In Africa, the Republic of Congo [28] and Gabon [29] also shows differences when compared with these Angolan data, except in the province of Cabinda. The differences found between Cabinda isolates and the rest of studied isolates may be due to geographical location of this province and its proximity to the neighboring countries Gabon and Congo and the movement of people between these regions. Most of the *P*. *falciparum *isolates (91,1%) revealed the haplotype 437**G**540**K **for *pfdhps *gene with low prevalence of mutation at codon 540 (3,2%).

The triple *pfdhfr*/*pfdhps *(59**R**108**N**/437**G**) mutant haplotype was found in 27% of isolates, the 51**I**108**N**/437**G **was the mutant haplotype more prevalent in studied isolates.

The results obtained in the present study are in line with those obtained with Iranian isolates where 51**I **mutation seems be a good molecular marker for the triple mutant *pfdhfr/pfdhps *[47]. By the other hand, theses results seem to be in contrast with the data obtained in a study carried out in Mozambique which claimed that the mutations at codon 59 in *pfdhfr *and codon 437 in *pfdhps *were enough to predict SP treatment failure [48], as well as in Burkina Faso [49] where the results showed that *pfdhfr *59**R **is more relevant than the 51**I **as a marker of SP treatment failure.

Finally, this is the first molecular study carried out in Angola including a large number of samples (452) from five different provinces of the country, and where five mutations of *pfdhps *and *pfdhfr *genes, predictive of SP therapeutic failure were screening showing high frequencies of 51**I**, 108**N ***pfdhfr *alleles, and 437**G **of *pfdhps *gen*e *with an almost absence of the quintuple mutation for SP.

## Conclusion

The high frequencies of mutations and haplotypes linked to antimalarial treatment failure (for example the 88% frequency of isolates carrying *pfdhfr *51**I**108**N **- *pfdhps *437**G **triple mutant allele, critical to SP resistance) herein described, highlight the need to revaluate the strategy of SP introduction as an IPT in children as well as the current use of SP for pregnant women IPT purposes, in Angola.

## Competing interests

The authors declare that they have no competing interests.

## Authors' contributions

FF coordinated sample collection. FF, RD carried out the selection of children and sample collection. RD, ZN and DL carried out DNA extraction and *Plasmodium *species identification. PF carried out the molecular analyses. FF, VEdR and DL coordinated and designed the study. DL drafted this manuscript. All authors read and approved the final manuscript.
